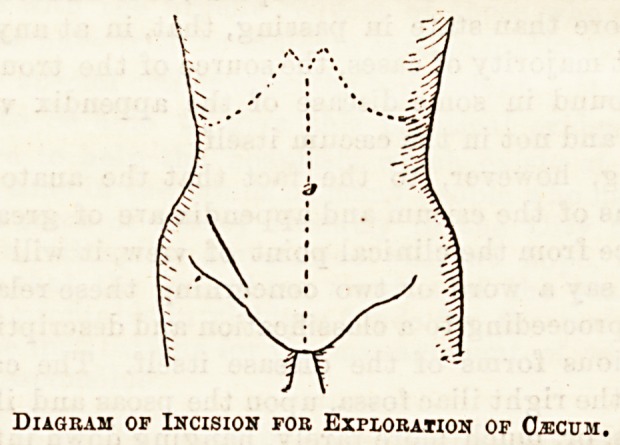# Perityphlitis

**Published:** 1895-09-21

**Authors:** E. Percy Paton


### MEDICAL PROGRESS AND HOSPITAL CLINICS.

[*The Editor will be glad to receive offers of co-operation and contributions from members of the profession. All letters should be addressed to* THE EDITOR, THE LODGE, PORCHESTER SQUARE, LONDON, W.]

### PERITYPHLITIS.

By E. PERCY PATON, M.D., M.S., F.R.C.S.

The term perityphlitis is a clinical and not a pathological one, and is applied to inflammation occurring around the cæcum, and as I propose to deal with it merely from the clinical standpoint, it is unnecessary to do more than state in passing, that, in at any rate the vast majority of cases, the source of the trouble is to be found in some disease of the appendix vermiformis, and not in the cæcum itself.

Owing, however, to the fact that the anatomical relations of the cæcum and appendix are of great importance from the clinical point of view, it will be as well to say a word or two concerning these relations before proceeding to a classification and description of the various forms of the disease itself. The cæcum lies in the right iliac fossa, upon the psoas and iliacus muscles, or, much more rarely, hanging down into the pelvis; its apex usually corresponds to about the middle of Poupart's ligament. Much the most important point about its relations, however, is the fact that it is entirely enveloped in peritoneum, as is also its appendix. This fact is of great moment, as upon it very largely depend the symptoms and treatment of the disease under discussion, which is always a form of peritonitis. The idea of the existence of a paratyphlitis—that is, an inflammation of the connective tissue round the cæcum—has now been abandoned, as it has been shown that there is no such collection of connective tissue.

Classification: From a clinical point of view there are three main varieties of the disease, namely—(1) Simple perityphlitis, (2) perityphlitic abscess, and (3) acute general peritonitis. Each of these will now be described.

**I. Simple Perityphlitis.**—The essence of the disease is a local adhesive or plastic peritonitis.

Symptoms and cause: The onset is usually more or less sudden, often absolutely so, the first complaint being of pain, which may be very acute, situated over the cæcum, and often accompanied by nausea and vomiting. This is followed by complete constipation in most cases, and some, often very considerable abdominal distension. Tenderness over the cæcum is always very markedly present, and the temperature is raised several degrees. The attitude of the patient is often very suggestive. He lies on his back, with the right leg drawn up and supported by the left leg, so, as far as may be, to relax the abdominal wall. Examination of the abdomen reveals the distension above mentioned; but also, what is of much more importance, a fulness in the right iliac fossa. This fulness is often of a more or less doughy consistency with ill-defined limits; but sometimes the limits are more clearly defined, and merely a mass like a bantam's egg may be felt. The mass is always very tender, a special point of tenderness being often noticeable situated on a line joining the anterior superior iliac spine and the umbilicus, about one and a half to two inches from the former. This is known as McBurney's point.

The course of this form of the disease is towards recovery. Under appropriate treatment, which will be described later, the urgency of the symptoms gradually disappears, though the mass may increase for the first few days or weeks; the gradual disappearance of tenderness being the most satisfactory sign of resolution. In about three weeks, or in a mild case less, the patient may be practically well again, the most important indication being the absence of all tenderness. Ill-defined thickening may be left for some time longer, or sometimes also a better defined mass like the end of one's thumb. This later is unsatisfactory if it remains, as it often indicates a tendency to relapse, of which further mention will be subsequently made.

**II. Perityphlitic Abscess.**—This frequently begins in a manner which is quite undistinguishable from the simple variety above described, and is only differentiated by its subsequent course. Even the temperature in the earlier stages gives no clear indication that pus is in the process of formation, except from the fact that, instead of in a comparatively short time the chart showing a gradual and satisfactory fall, the temperature usually remains obstinately raised. Rigors or even shivering, so often present under similar conditions elsewhere, seem rarely to be present in these cases. The indication of pus, on which most reliance must be placed, is the continuous increase instead of recession of the symptoms and signs, and not usually any distinctive difference in their character. Of course, if time be given, redness, œdema, and even fluctuation will appear, though the last is very late in occurring, and waiting for these signs is a most unwise delay. It may be a week or two before pus is clearly present in some cases; but in other much more acute ones there may be a fair collection by the end of the first week. Usually, more especially in England, where early operation is not so much in vogue as in America, incision is rarely considered necessary before the third week. Some cases, however, are much more chronic and slower to develop than this. Since the pus is a localised peritoneal abscess, enclosed by adhesions, the great danger is that these adhesions may give way, resulting in a sudden general peritonitis. Another complication of this form of the disease is swelling of the leg, due to spread of the inflammation to, or pressure upon, the iliac vein, which is in close contiguity to the seat of inflammation.

**III. Acute General Peritonitis.**—This generally follows some form of exertion, which causes rupture of the appendix, which has previously been diseased, without giving any indication that it was so.

The symptoms are very acute, often so much so that it can only be surmised that the cæcum is the source of the trouble, except from the fact that the most acute tenderness and the spread of the disease seems to be from the right iliac fossa. All the symptoms of very virulent peritonitis are soon obvious, such as pain distension, tenderness, collapse, absolute constipation, and vomiting, and the tendency of the disease is towards a rapid, fatal ending in the course of a few days.

A few words must now be said about cases of what is called "relapsing typhlitis." These are cases in which an ordinary attack of pericæcal inflammation is followed, after a shorter or longer interval, by a second similar attack, and this again by a third, and so on, unless in one of them suppuration sets in, necessarily forcing active treatment. It is easily to be seen how a fairly rapid succession of such illnesses may so weaken the patient as to ultimately lead to a fatal result or to chronic invalidism.

The cause of them has been clearly shown to be persistent disease of the appendix, which breaks out in active surrounding inflammation on the least provocation, such as exposure to cold or indiscretion in diet. In these cases, even when the active symptoms have quite subsided, an enlarged and somewhat sensitive appendix can sometimes still be made out by a careful palpation of the right iliac fossa.

**Diagnosis** of the disease in its various forms may be very easy or very difficult. In the ordinary and more common simple cases the difficulties are usually slight; the pain and tenderness with the mass in the iliac fossa and the accompanying symptoms leaving no room for doubt. In cases which are less clear, especially in women, it may be doubtful whether some pelvic inflammation such as periovaritis may not be the trouble present. Careful examination of the pelvic organs per vaginum and per rectum will, however, usually clear this up. It must not, however, be forgotten that sometimes an enlarged appendix can be felt per rectum. Ileo-cæcal intussusception also must be kept in mind, though in this some characteristic symptoms, such as tenesmus, the passing of blood and slime, and the movement of the tumour as the intussusception increases, and its general contour will usually clear up the case. The most difficult cases of all are those very acute ones, especially with considerable abdominal distension and absolute constipation which simulate so exactly acute bowel obstruction. It is only by carefully weighing all the features of the case that a correct opinion can be come to, and sometimes the nearest that can be reached is that the case is one of obstruction or pericæcal disease.

**Treatment.**—This may be described as of two kinds, the expectant and the active. The former consists in the ordinary treatment of a peritoneal inflammation by rest in bed, opium, and the blandest of fluid diets, which should at first err on the side of being too limited than too excessive in quantity. Local applications of belladonna and glycerine, in combination with moist heat, should be used over the tender region to relieve pain. The bowels should not be disturbed for a day or two until the acutest symptoms have subsided, and then they should be solicited by an enema. A saline purge may be given as soon as the inflammatory symptoms have quite quieted down. The patient should be kept on a restricted diet, and in bed, until all tenderness has quite gone. This line of treatment is suitable for all the simple cases, which do not go on to suppuration. Should, however, signs of the formation of pus be present, such as the temperature keeping up, and swelling in the iliac fossa still continuing to increase after the first few days, an incision must be made, this being very much to be preferred, even when there is some slight doubt as to the presence of pus, than the use of the exploring needle. The incision recommended by Treves is one made obliquely from above downwards, ending a little above and outside the middle of Poupart's ligament. When the pus is reached the cavity should be washed

Diagram of Incision for Exploration of Cæcum.

out, but with great gentleness, as it is to be remembered that the pus is in the peritoneal cavity, only shut off from it by adhesions. If the appendix be easily seen it may be cut off after the stump has been tied with silk or gut. No great search, however, should be made for it. A drainage tube is then put in, and the wound partly closed. Subsequent treatment is as for an ordinary abscess.

In the very acute cases, which are really cases of acute general peritonitis, the only hope is a free opening into the peritoneum over the cæcum, the appendix being, if possible, found and clamped above the perforation in it, and then cut off, part of its peritoneal covering being first turned back as a kind of cuff. This is then invaginated, and one or two Lembert's sutures put in to close it up. If speed is a consideration, merely placing a ligature round the stump will be sufficient. The peritoneum must now be irrigated freely with warm water, especial attention being paid to the pelvis and the hollows in the flanks. The fluids are then sponged out, and the abdomen closed with or without a drain, according to the surgeon's discretion.

The treatment of relapsing cases is a recognition of the fact that the recurrence is due to persistent disease of the appendix. An acute attack should, therefore, be treated on ordinary expectant principles; but when a quiescent period has been reached an incision is made in the position described above for opening a perityphilitic abscess, or, better still, over the enlarged appendix if its situation can be made out by palpation. It may now be easy to reach the diseased organ, or a careful and difficult dissection through close adhesions may be necessary to expose it. It should then be clamped and cut off, and its stump secured by sutures in the manner above described, the general abdominal cavity being during this time carefully guarded by sponges. The abdomen is then closed without a drain. These cases, almost without exception, do extremely well if done, as described, during a quiescent and not an inflammatory period.

## Figures and Tables

**Figure f1:**